# Universities Scale Like Cities

**DOI:** 10.1371/journal.pone.0059384

**Published:** 2013-03-27

**Authors:** Anthony F. J. van Raan

**Affiliations:** Centre for Science and Technology Studies (CWTS), Leiden University, Leiden, The Netherlands; University of Maribor, Slovenia

## Abstract

Recent studies of urban scaling show that important socioeconomic city characteristics such as wealth and innovation capacity exhibit a nonlinear, particularly a power law scaling with population size. These nonlinear effects are common to all cities, with similar power law exponents. These findings mean that the larger the city, the more disproportionally they are places of wealth and innovation. Local properties of cities cause a deviation from the expected behavior as predicted by the power law scaling. In this paper we demonstrate that universities show a similar behavior as cities in the distribution of the ‘gross university income’ in terms of total number of citations over ‘size’ in terms of total number of publications. Moreover, the power law exponents for university scaling are comparable to those for urban scaling. We find that deviations from the expected behavior can indeed be explained by specific local properties of universities, particularly the field-specific composition of a university, and its quality in terms of field-normalized citation impact. By studying both the set of the 500 largest universities worldwide and a specific subset of these 500 universities -the top-100 European universities- we are also able to distinguish between properties of universities with as well as without selection of one specific local property, the quality of a university in terms of its average field-normalized citation impact. It also reveals an interesting observation concerning the working of a crucial property in networked systems, preferential attachment.

## Introduction

Recent work of Bettencourt and West and their colleagues on urban scaling [1], [2] addresses the behavior of cities as complex systems. The most surprising discovery [3] is that size (in terms of population, number of inhabitants) is the major determinant of most characteristics of a city; history, geography and design have secondary roles. As a result of nonlinear interaction in socioeconomic dynamics, an increase of population size of cities (urban agglomerations ranging from about 100,000 to 10,000,000 inhabitants) leads to a disproportional, power law scaling of important indicators such as economic productivity and innovation capacity, for instance the GMP, the Gross Metropolitan Product. The power law exponents are around 1.15. This means that with every doubling of population, regardless of a city’s initial size, the increasing returns to scale (population size) is about 15%. Thus, larger cities produce wealth and new ideas faster. The underlying mechanism is that social interactions become more effective with city size: more economic specialization and division of labor, but at the same time more interconnections and increasing advantages in greater economics of scale, particularly in all kinds of city infrastructure. The power law scaling of socioeconomic city indicators with population size provides the expected behavior. Local characteristics of a city however may cause deviations from the expected behavior.

In this paper we demonstrate that universities show a similar behavior as cities. Universities too are complex systems with their own diverse but interrelated infrastructural facilities, social and economic characteristics. Instead of the Gross Metropolitan Product we use the ‘gross university income’ in terms of total number of citations; instead of city defined size by population we use university size in terms of total number of publications. We find that deviations from the expected behavior can indeed be explained by local properties of universities.

Earlier relevant work was done by Katz [4], [5], [6] who discussed scaling relationships between number of citations and number of publications across research fields and countries. He concluded that the science system is characterized by a size-dependent cumulative advantage. Cumulative advantage refers to any process in which a specific quantity, mostly a measure of wealth or credit (in our case: citations), is distributed among objects (in our case: publications) according to how much they already have: “the rich get richer”, commonly known as the ‘Matthew effect’ [7], [8], [9]. The term cumulative advantage was coined by De Solla Price [10]. In network theory the same process is nowadays referred to as preferential attachment: the more connected a node is, the more likely it is to receive new links. In a general sociological context it means that an internationally renowned, prolific scientist will often get more credit (for instance, in terms of citations) than a comparatively less renown scientist for work of similar quality. In the context of this study the cumulative effect implies a nonlinear increase of impact with increasing size, demonstrated by the finding that the number of citations as a function of number of publications exhibits a power law dependence with an exponent larger than 1. In our previous articles [11]–[13] we demonstrated a size-dependent cumulative advantage of the correlation between the number of citations and the number of publications at the level of research groups and on the level of universities [14]. In a broader perspective, our work is related to the study of the emergence of scaling in networks characterized by preferential attachment and growth [15]. Important progress has been made by studying networks on the basis of time-resolved large-scale data. Real life examples are networks of collaborators [16], citations [17], and words [18].

Recent work on scaling properties of European research units [19] showed a power law scaling relation between the number of support staff and the number of academic staff. Because the number of academic staff and the number of publications of research units are generally strongly related, this finding suggest a power law relation between scientific size and administrative size. Translated to cities, this could imply a power law scaling between population size and the size of urban bureaucracy.

## Results and Discussion

### Data and Method

We used the data of the 500 largest universities in the world in terms of publications covered by the Web of Science which we collected for the Leiden Ranking 2011–2012 [20]. The Web of Science (WoS) is the comprehensive multidisciplinary publication and citation database produced by Thomson Reuters. Our institute (CWTS) has built an improved and enriched version of the WoS for bibliometric studies. Another large multidisciplinary publication and citation database is Scopus produced by Elsevier. The Leiden Ranking 2011–2012 is based on publications in the period 2005–2009, and citations to these publications in the period 2005–2010. There will be an update of the Leiden Ranking in April 2013.

For our analysis we used the entire data set of the 500 universities worldwide, and a subset of the 100 European top-universities. In this way, we may find differences in the deviations from the expected behavior between the whole set and a subset of universities that are selected on the basis of quality (in terms of citation impact). For a brief but comprehensive and clear explanation of the bibliometric indicators we refer to the methodology section of the Leiden Ranking website [20] and to our recent paper on data collection, indicators, and interpretation of the ranking [21]. The Leiden Ranking supports two counting methods: full counting and fractional counting. The full counting method gives equal weight to all publications of a university. The fractional counting method gives less weight to collaborative publications than to non-collaborative ones. For instance, if the address list of a publication contains five addresses and two of these addresses belong to a particular university, then the publication has a weight of 0.4 in the calculation of the bibliometric indicators for this university. The fractional counting method leads to a more proper normalization of indicators and to fairer comparisons between universities. Fractional counting is therefore regarded as the preferred counting method in the Leiden Ranking.

We also distinguish between the options ‘all’ WoS covered publications and ‘English publications only’. Comparing the impact of non-English language publications with the impact of publications written in English may not be considered fair. Non-English language publications can be read only by a small part of the scientific community, and therefore these publications cannot be expected to receive similar numbers of citations as publications written in English. Particularly for German and French universities this has a strong negative effect on their citation impact and with that on their ranking positions [22], [23]. Therefore the Leiden Ranking 2011–2012 offers the possibility of excluding non-English language publications from the calculation of the bibliometric indicators next to using all publications. In this study we applied the fractional counting method for English-language papers.

First we analyzed the entire set of 500 universities worldwide. We took the following approach. We determined the correlation between the absolute number of citations *C*, considered as the ‘gross university income’, and the absolute number of publications *P*, considered as ‘size’ of a university. This yields the average scaling behavior of *C* for a university with size *P*. Next, we determined the deviations of from this average for each individual university by calculating the residuals of the scaling distribution in a similar way as Bettencourt *et al* (2010) did for urban scaling. Finally, we investigated with which bibliometric indicators the residuals correlate. The same procedure is followed with a slightly different scaling in which the number of citations is replaced by the field-normalized number of citations.

### 500 Largest Universities Worldwide

**Figure 1:**
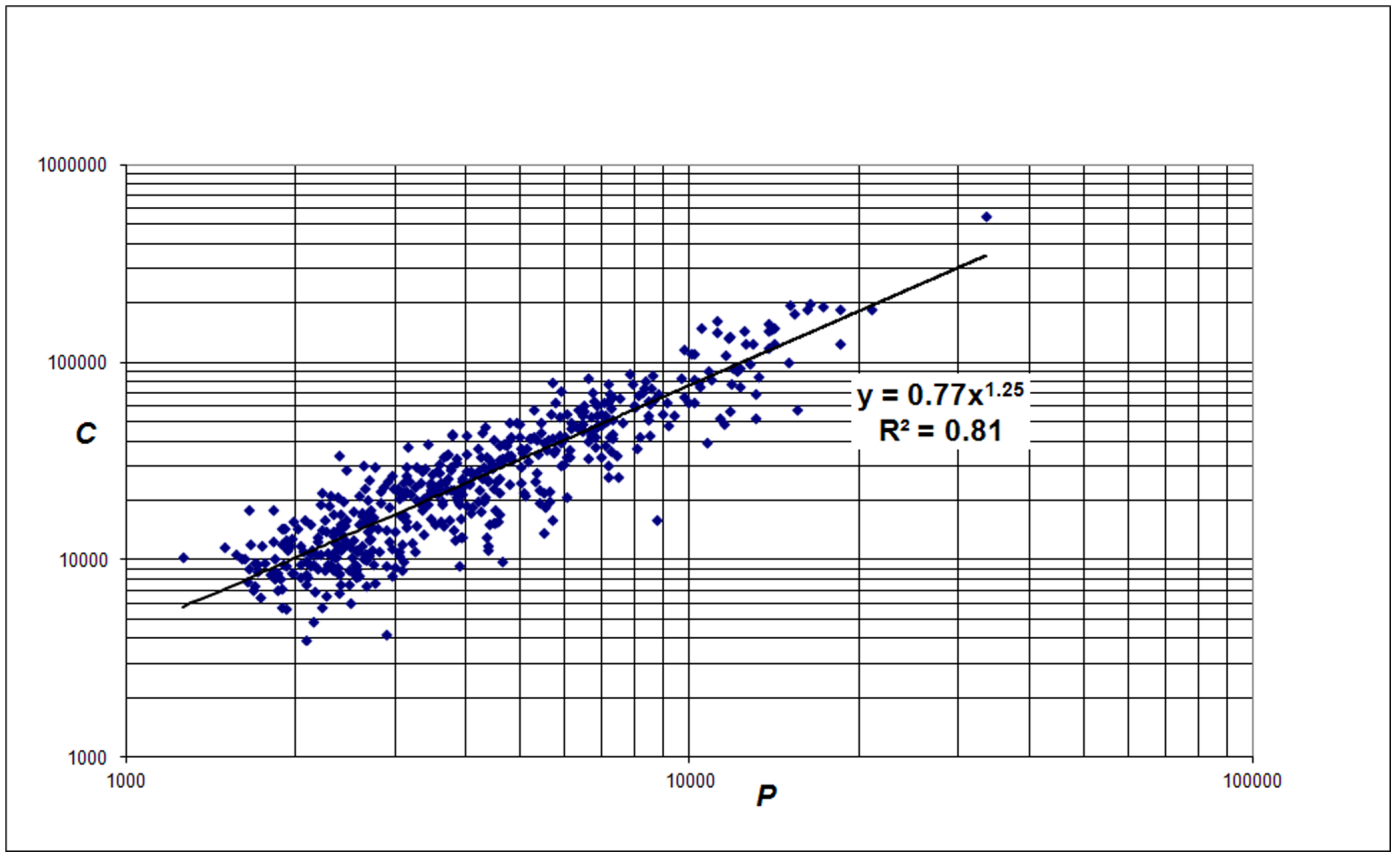
Correlation of *C* (citations without self-citations, ‘income’) with *P* (‘size’) for the 500 largest universities worldwide.

The first analysis is carried out with the entire set of the 500 largest (in terms of WoS covered publications) universities worldwide. These 500 largest universities can be found in the Leiden Ranking site [20]. We find a power law relation between citations and publications of a university, i.e., a nonlinear relation given by
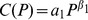
(1)with exponent *β_1_* = 1.25, see Fig. 1. The coefficient *a_1_* with value 0.77 is a normalization constant. As discussed above also on lower aggregation levels, particularly research groups, we find similar scaling properties [11]–[13]. We found that in the case of full counting and all papers (so not only the English-language papers) the exponent is 1.30.

Denoting the observed value of the number of citations for each specific university with *C*_i_ we calculated the residuals of the scaling distribution of each university in a similar way as in the urban scaling study [2]:
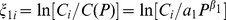
(2)


**Figure 2:**
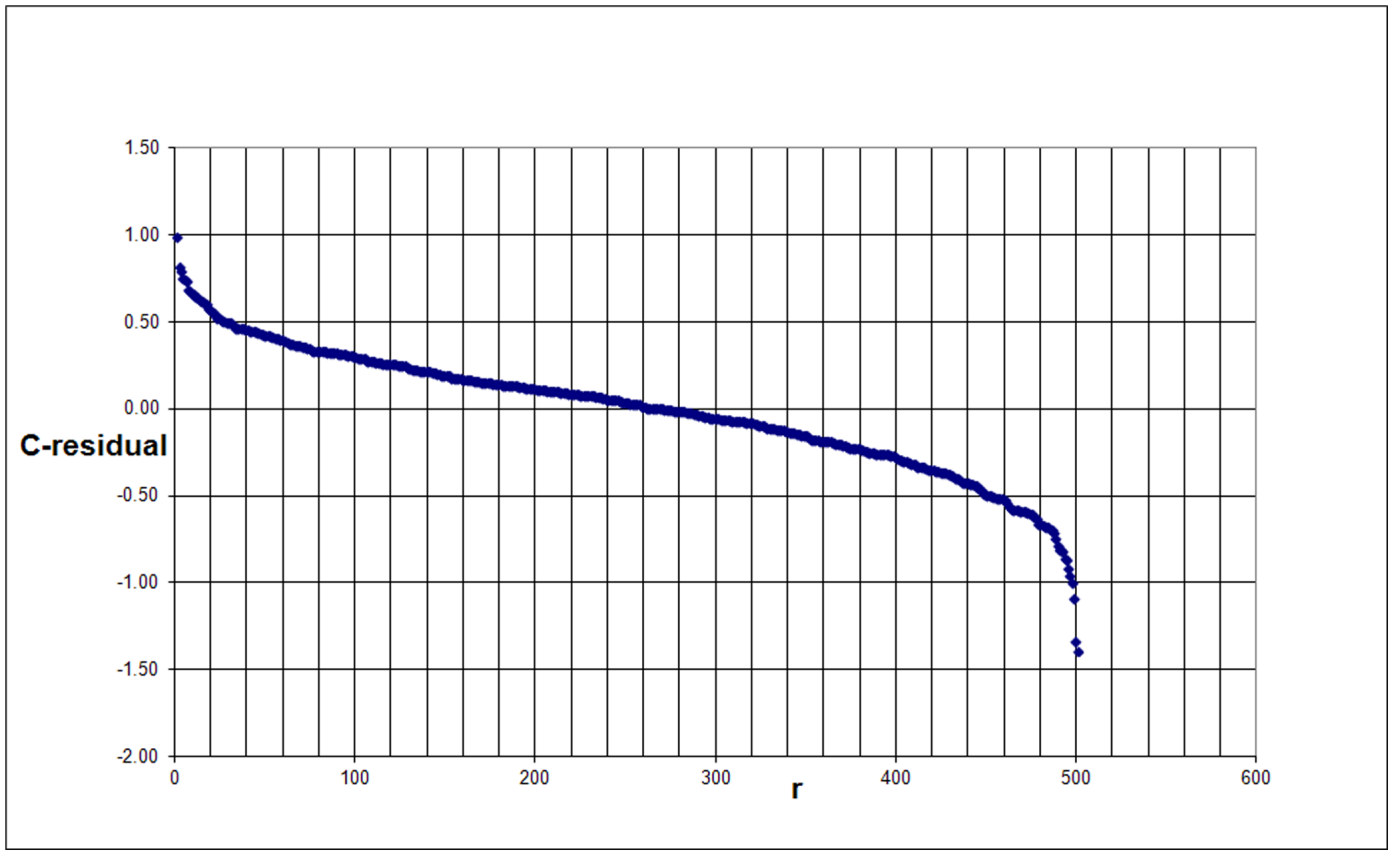
Ranking of the C-residuals of the 500 universities.

Because we will also calculate the residuals of a slightly different scaling, we call the above residuals the C-residuals. The residuals are a measure of the deviation of the real (observed) from the predicted number of citations for each university. In Fig. 2 we show the ranking of the C-residuals for the 500 universities.

**Figure 3:**
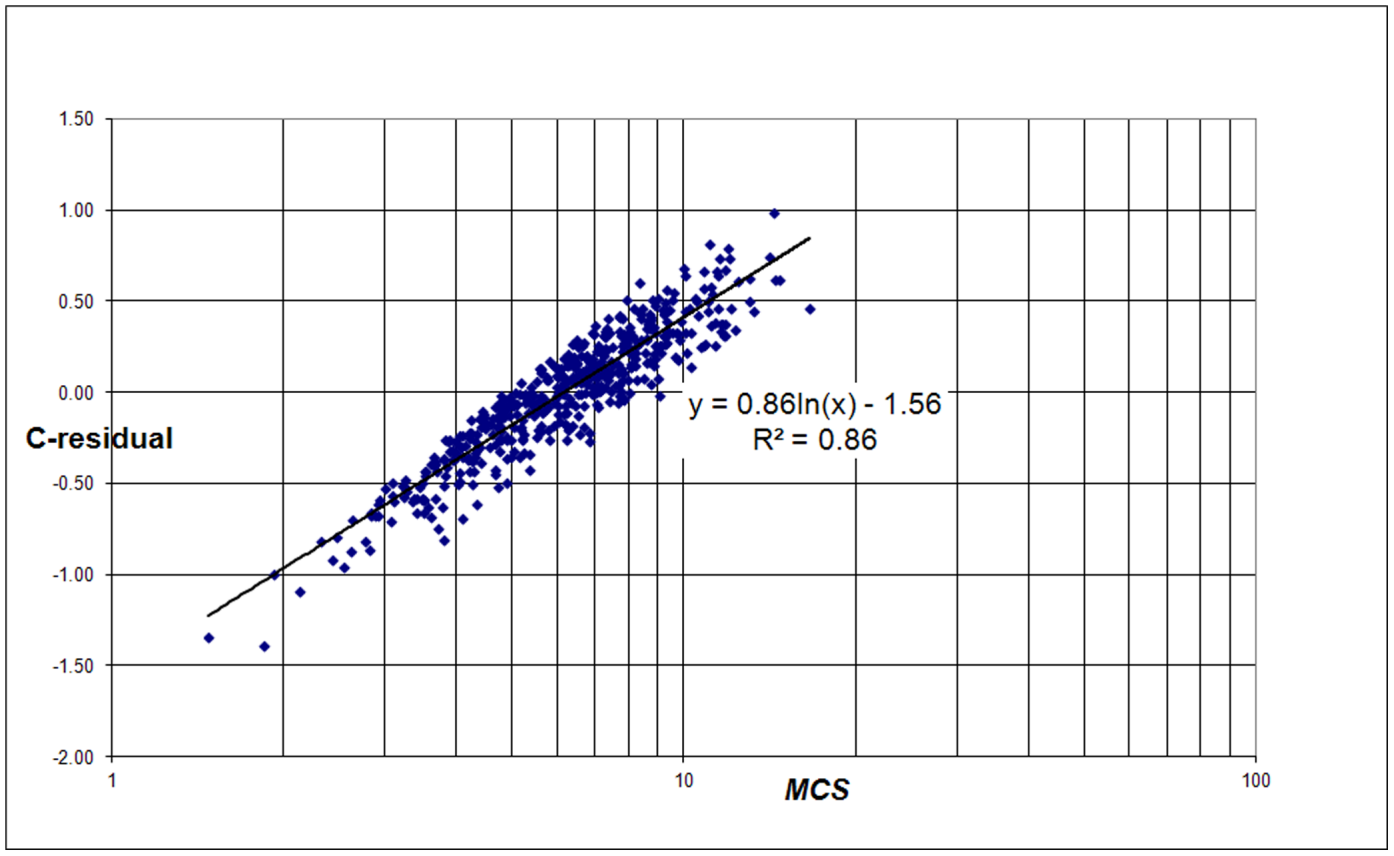
Correlation of the C-residuals with the average number of citations per paper for each university (*MCS*) for the 500 largest universities worldwide.

**Figure 4:**
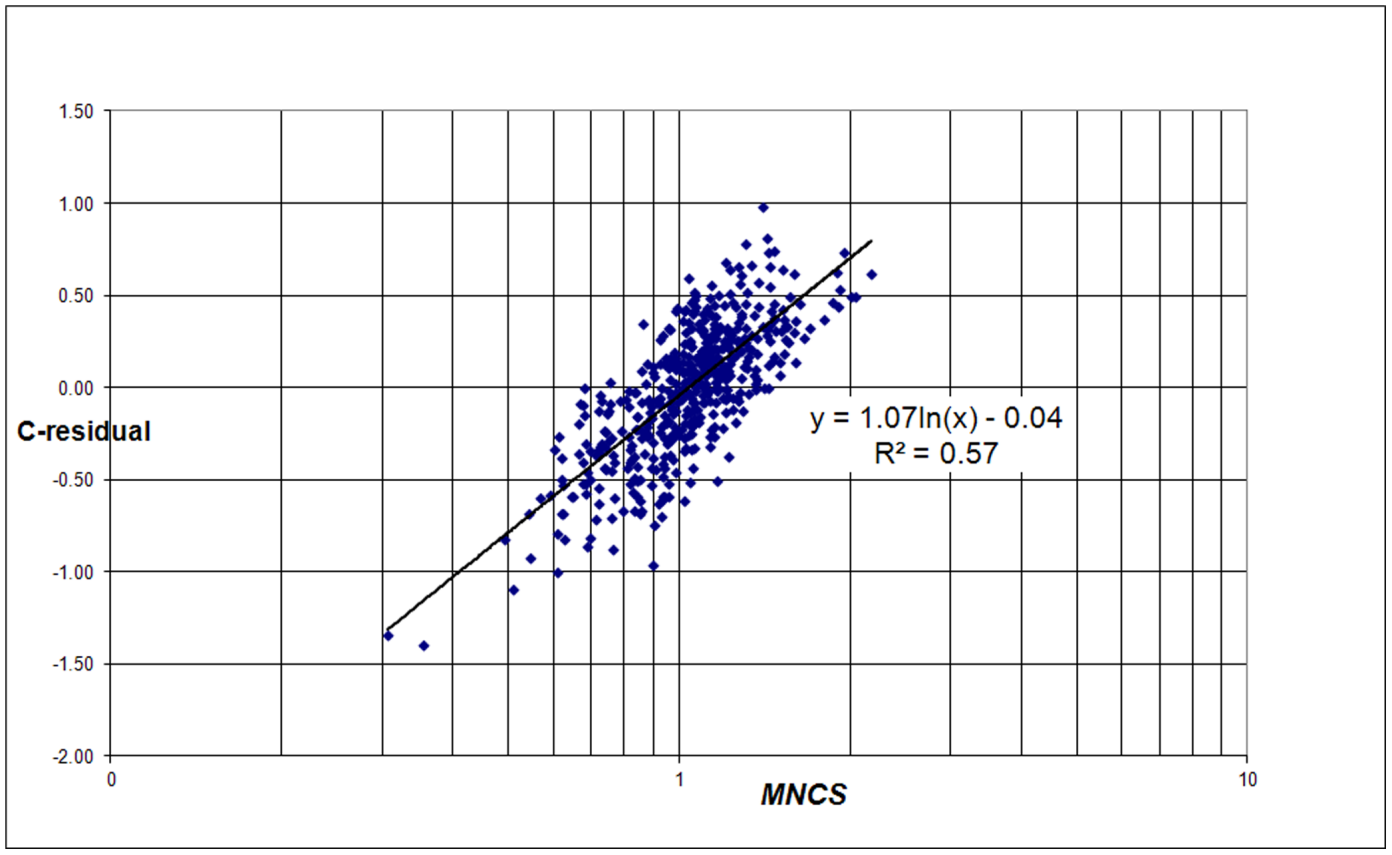
Correlation of the C-residuals with the average number of field-normalized citations for each university (*MNCS*) for the 500 largest universities worldwide.

The central question is what causes the deviations. Particularly, which universities have positive while other have negative residuals. Therefore our next step is to find out with which bibliometric indicators the residuals correlate. We first analyzed for each university the relation with the average number of citations per paper (Mean Citation Score, *MCS*) and with the average *field-normalized* number of citations (Mean Normalized Citation Score, *MNCS*) of a university, see Figs. 3 and 4. The *MNCS* is also normalized for document type (such as letters, reviews or ‘normal’ papers). An *MNCS* value of two for instance means that the publications of a university have been cited twice above world average.

Notice that the correlation in both cases is not determined by a power law, but by a logarithmic relation. This is what we can expect given the definition of the residuals (see eq. 2). We find:
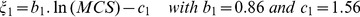
(3)and



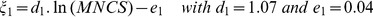
(4)The *MCS* indicator is simply citations per paper: it does not take into account the often large differences in citation density of the many scientific fields. As we showed in earlier work [14] the average number of citations correlates very strongly with the average field-citation density of a university. In other words, if a university is characterized by strong research activities in fields with a high citation density, and these are predominantly the (bio)medical and the basic natural science fields, then its *MCS* will be relatively high. In contrast, technical universities focus on engineering and applied research, in these fields the citation density is considerably lower as compared to the medical fields, and thus the average number of citation paper for these universities will be lower. The same is true for universities focusing on economics and social sciences.

**Figure 5:**
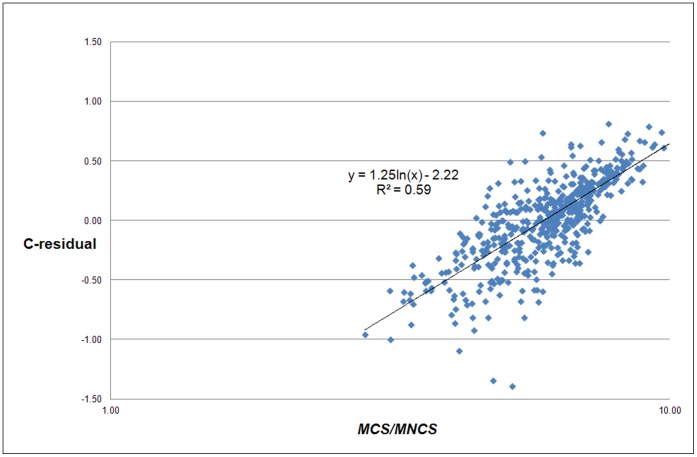
Correlation of the C-residuals with the average field citation-density for each university (*MCS/MNCS*) for the 500 largest universities worldwide.

However, the strong relation of the C-residuals with *MCS* is quite trivial: the C-residuals are a function of the real number of citations and the real number of publications of a university, and the same is true for *MCS*. If the power law exponent would be exactly 1, the correlation between the C-residuals and *MCS* would be perfect. Thus, we need a more convincing proof that the C-residuals are related with the field composition of a university. Therefore we analyzed the relation between the C-residuals and *MCS*/*MNCS*, see Fig. 5. Because *MCS* is the average number of citations per paper and *MNCS* the average field-normalized number of citations of a university, the ratio of both indicators is measure of the average field-citation density of a university, and hence a measure of its field composition.

Fig. 5 shows the dependence of the C-residuals on the average field citation-density and thus the field-composition of a university, which is a typical ‘local characteristic’. In line with our earlier discussion we expect that universities with a large medical school will generally have positive C-residuals, whereas universities with a strong focus on engineering or on social sciences will generally have negative C-residuals. Inspection of the lists of universities with positive and negative C-residuals confirms this assumption.

The *MNCS* indicator corrects for field density, it is the field-normalized impact of a university. As we see in Fig. 4, the C-residuals also correlate reasonably with *MNCS*. This implies that the residuals also reflect to some extent the research quality of a university (measured in terms of our field-normalized citation impact indicator).

**Figure 6:**
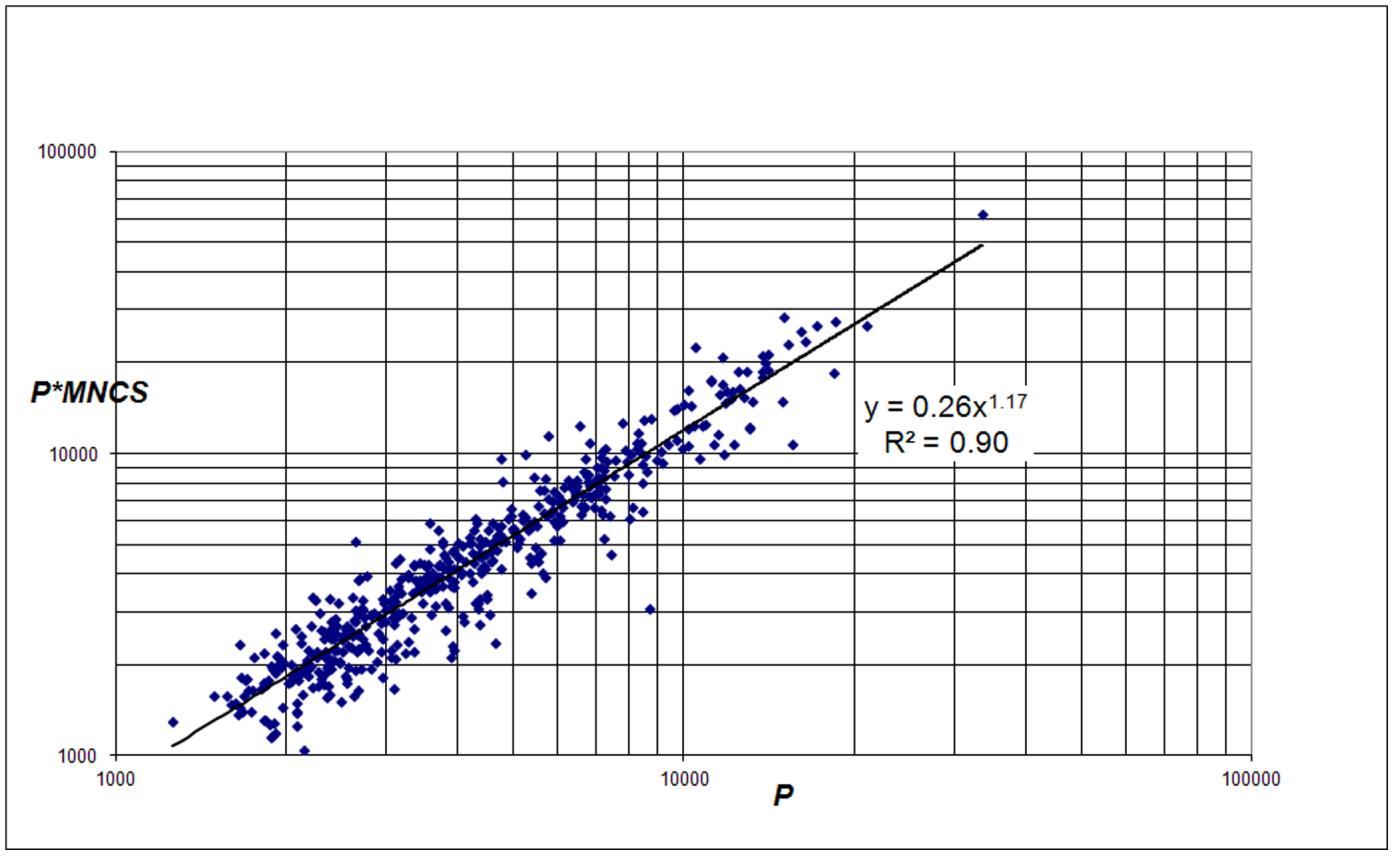
Correlation of *C*_n_ ( =  P*MNCS, the field-normalized number of citations) with *P* for the 500 largest universities worldwide.

In order to approach the problem of field-normalization in a slightly different way, we analyzed the correlation of the field-normalized absolute number of citations (*C*_n_ = P*MNCS) with the absolute number of publications (*P*). That P*MNCS is the field-normalized absolute number of citations can be seen in the following way: *MNCS* indicates the average field-normalized citation per publication and thus by multiplying with the total number of publications, the product is the field-normalized absolute number of citations. The results are shown in Fig. 6. We find a power law relation between field-normalized citations and publications of a university, i.e., a nonlinear relation given by
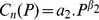
(5)with exponent *β_2_* = 1.17 and coefficient *a_2_* = 0.26 (for full counting and all papers the exponent is 1.19). We notice that the power law exponent in this case is lower than in the case of the non-normalized number of citations (eq. 1).

Also in this approach we calculated the residuals for each university:
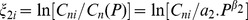
(6)


**Figure 7:**
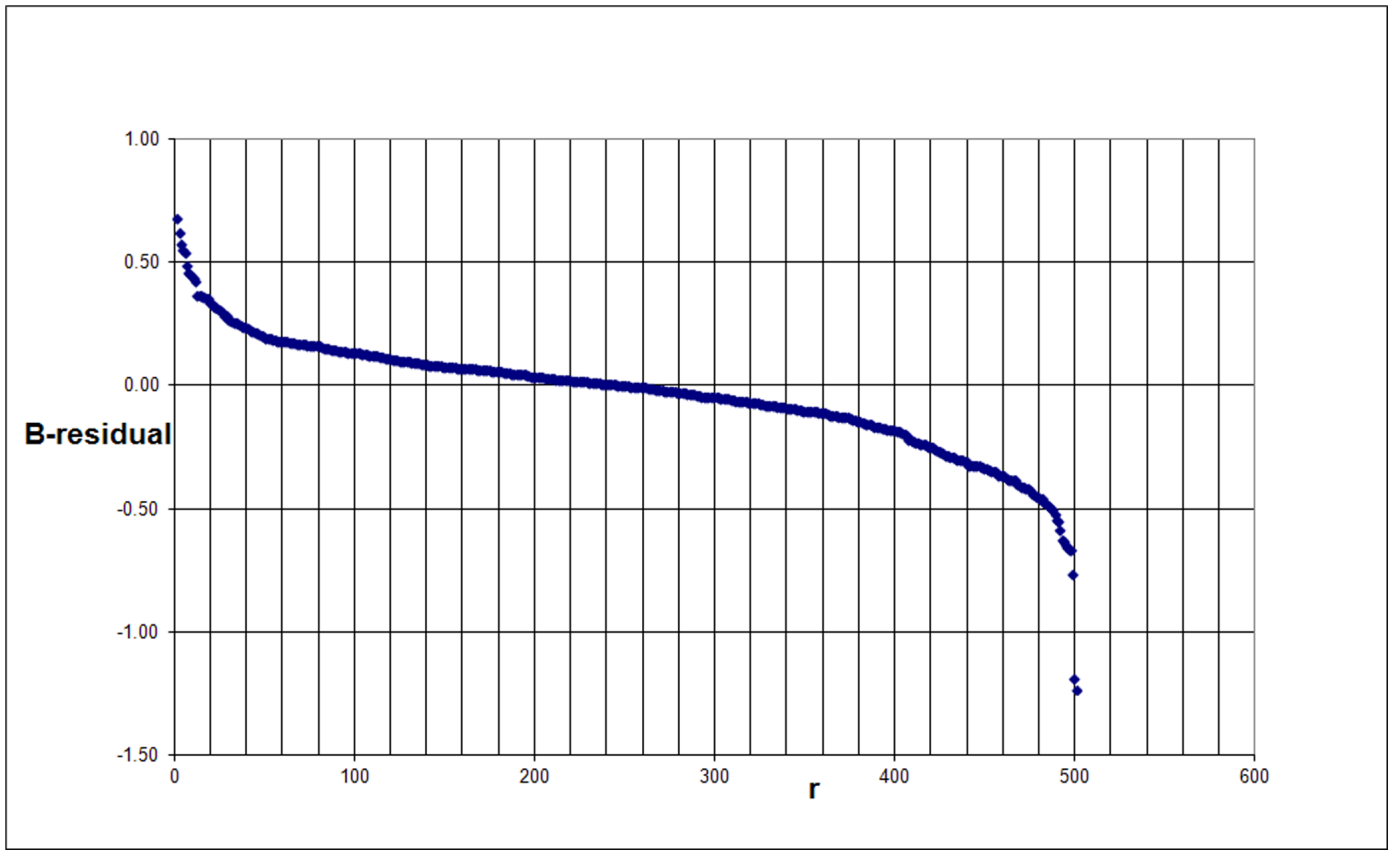
Ranking of the B-residuals of the 500 universities.

We call these residuals the B-residuals. In Fig. 7 we show the ranking of the B-residuals for the 500 universities. We see that the B-residuals cover a somewhat smaller range as compared to the C-residuals.

**Figure 8:**
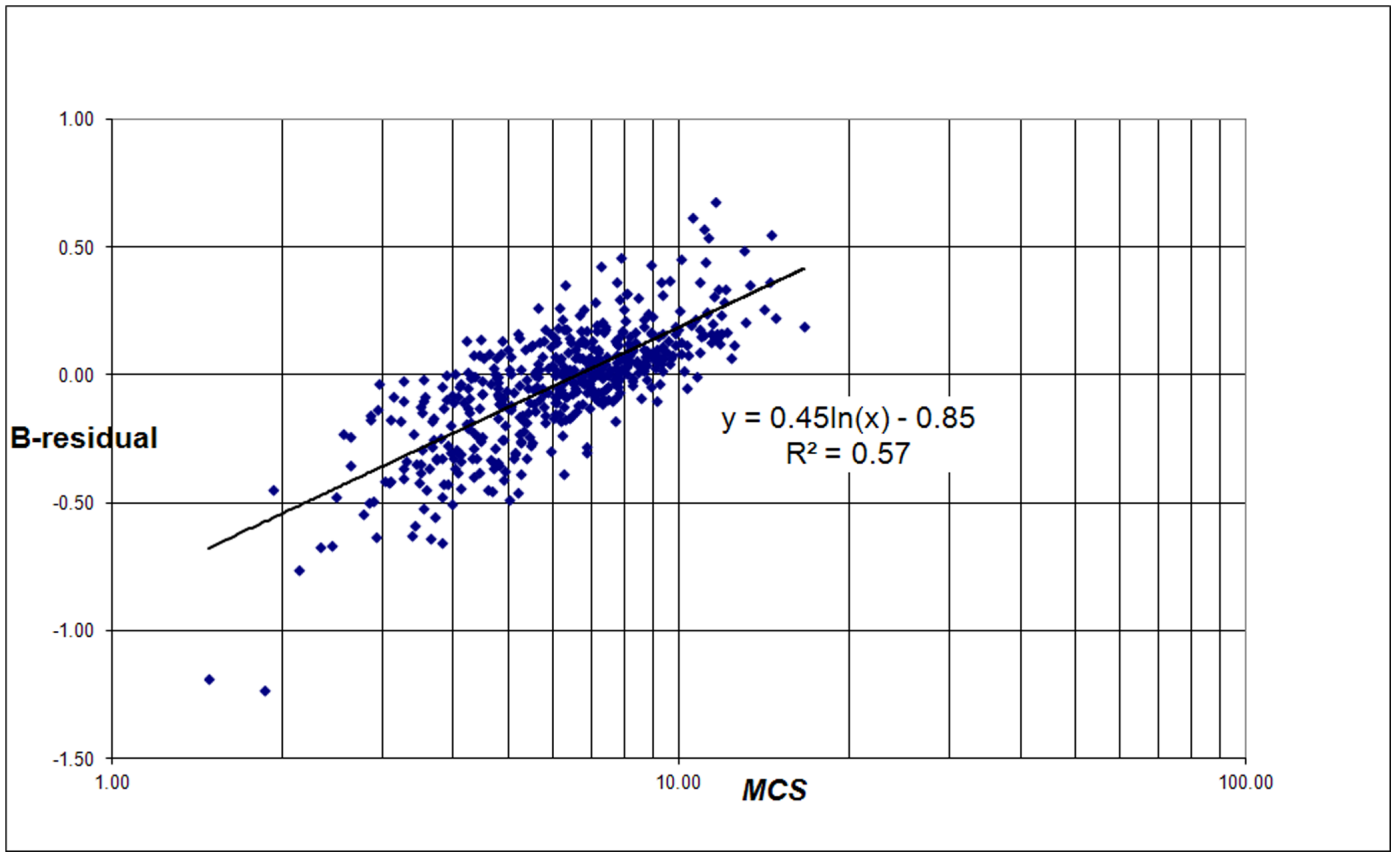
Correlation of the B-residuals with the average number of citations per paper for each university (*MCS*) for the 500 universities.

**Figure 9:**
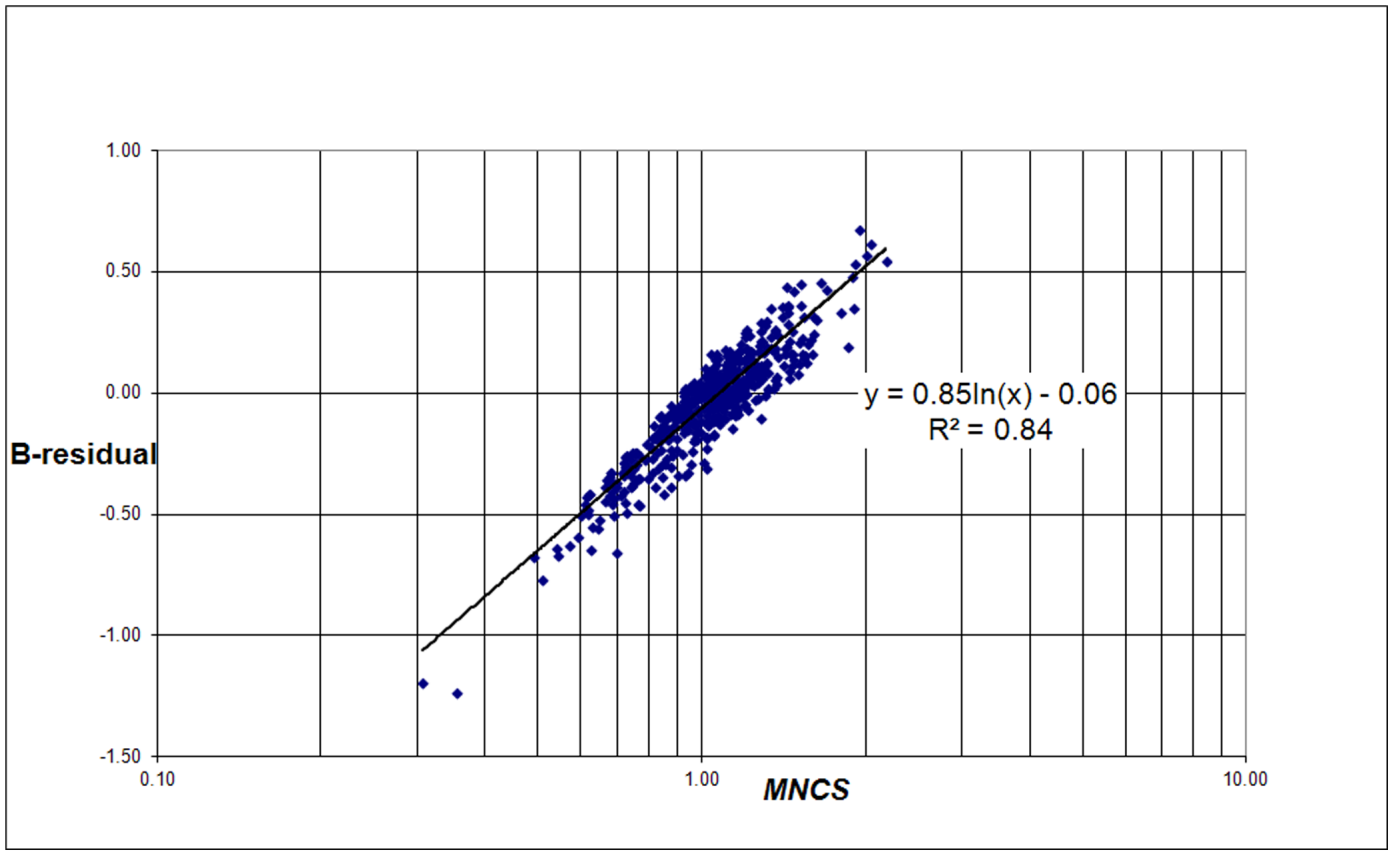
Correlation of the B-residuals with the average number of field-normalized citations for each university (*MNCS*) for the 500 universities.

The correlation of B-residuals with the average number of citations per paper (*MCS*) of a university and the average field-normalized number of citations (*MNCS*) of a university is shown in Figs. 8 and 9. Again we find a logarithmic relation:
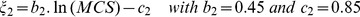
(7)and

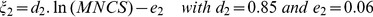
(8)


Because the B-residuals are field-normalized measures, we can expect that they will be sensitive for the non-normalized *MCS*. The B-residuals correlate stronger with *MNCS* than in the case of the non-normalized citation scaling. The reason is the same as for the strong correlation of the C-residuals with *MCS*: the B-residuals are a function of the real number of field-normalized citations and the real number of publications of a university, and the same is true for *MNCS*.

**Figure 10:**
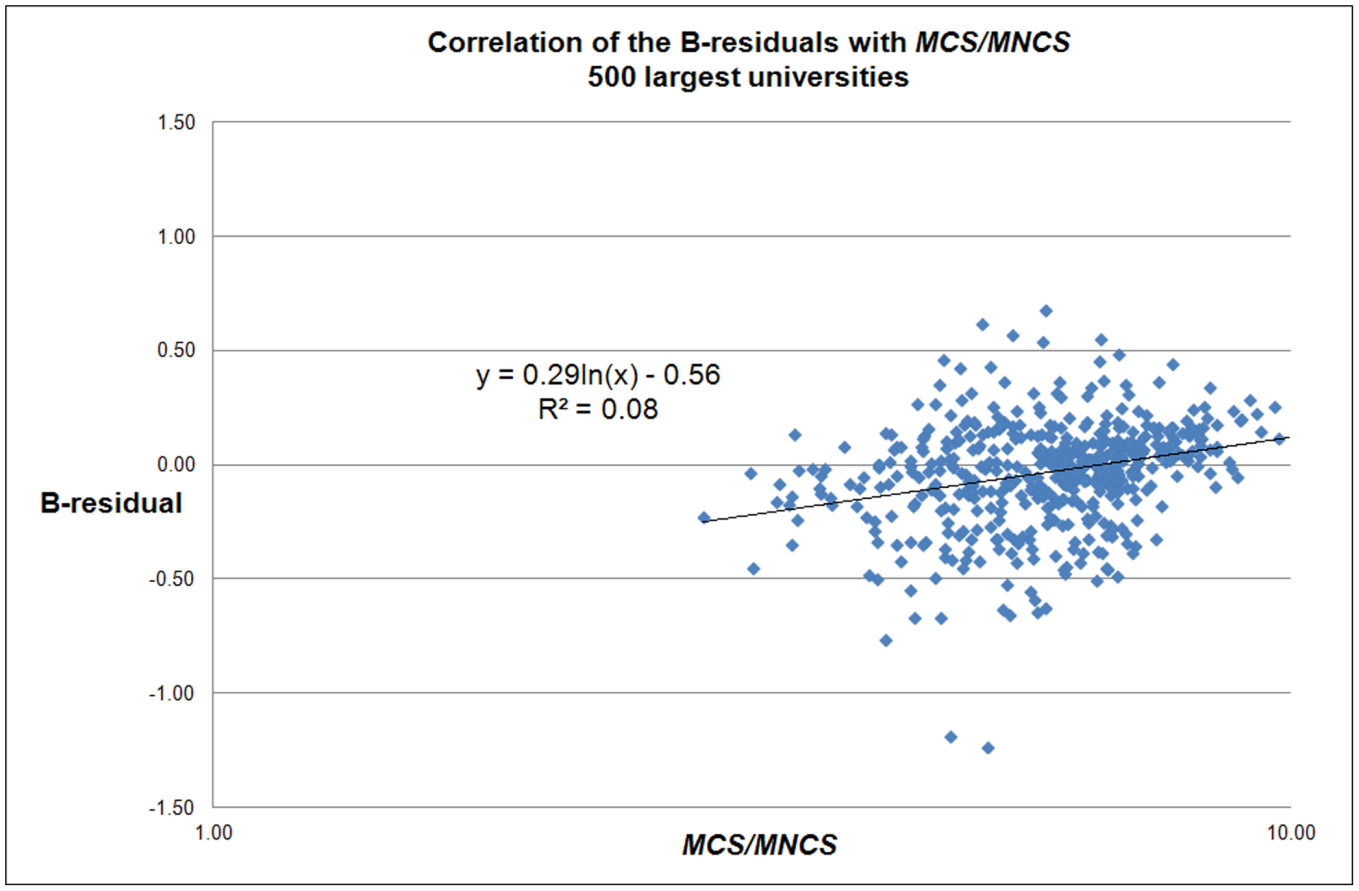
Correlation of the B-residuals with the average field citation-density for each university (*MCS/MNCS*) for the 500 largest universities worldwide.

There is no significant relation between the B-residuals and the field citation-density indicator *MCS*/*MNCS*, see Fig. 10. This is to be expected because the B-residuals are field-normalized.

**Figure 11:**
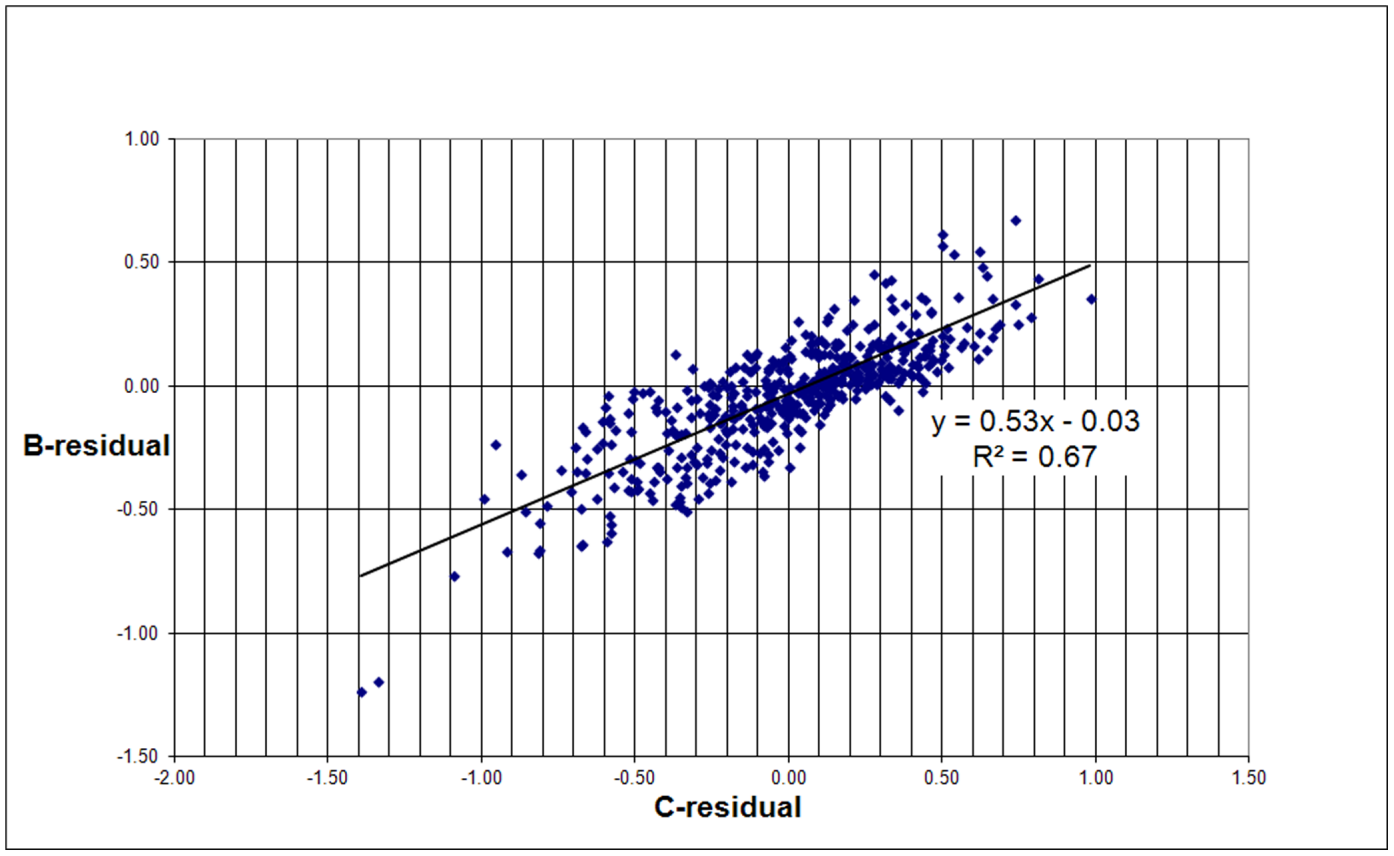
Correlation of the B-residuals with the C-residuals.

Finally, we analyzed the correlation between both types of residuals. As shown in Fig. 11, this correlation is quite significant.

As remarked earlier, we also performed the entire above analysis for the 500 universities with full counting and all WoS publications. We found that there are no significant differences in results for these two counting modalities.

### Top-100 European Universities

The selection of the 100 European top-universities was carried out as follows. From the 500 universities worldwide we first identified all European universities (in total 216 of the 500), ranked them by the *PPtop10%* indicator, and took the top-100. The *PPtop10%* indicator is the proportion of the publications of a university that, compared with other similar publications, belong to the top 10% most frequently cited of the field(s) to which the publication belongs. Publications are considered similar if they were published in the same field and the same publication year and if they have the same document type. In this way *PPtop10%* is a field-normalized indicator. With the selection tools provided in the Leiden Ranking site [20] one can easily find the top-100 European universities as defined in this paper.

**Figure 12:**
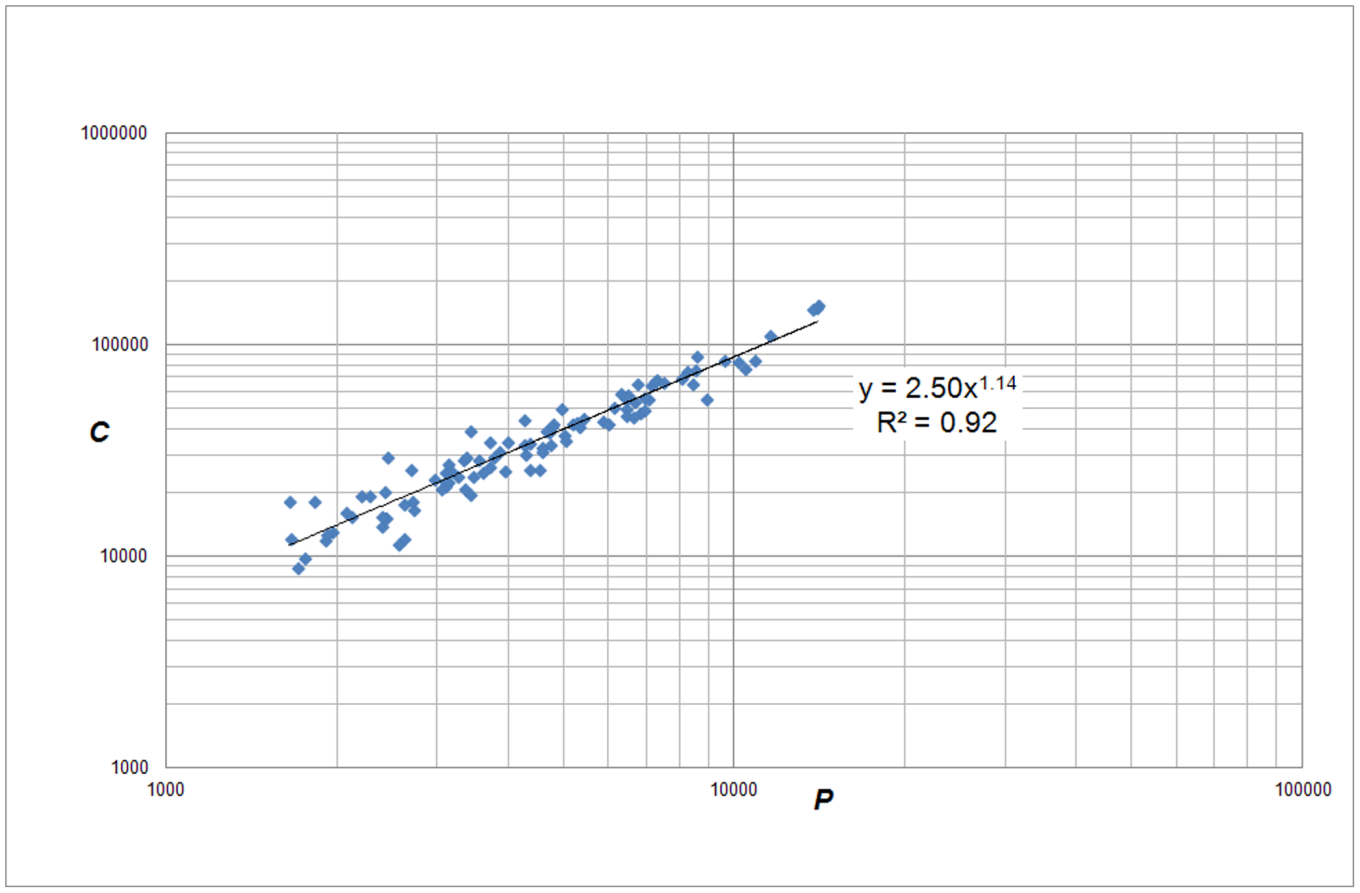
Correlation of *C* (citations without self-citations, ‘income’) with *P* (‘size’) for the top-100 European universities.

Again we find a power law relation between citations and publications of a university, but with a considerably lower exponent as compared to the 500 universities:
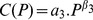
(9)with exponent *β_3_* = 1.14, and the coefficient *a* ***_3_*** = 2.50, see Fig. 12.

Our first observation is that for top-universities the nonlinear, cumulative advantage by size is considerably less as compared to the set of 500 universities without any quality selection. This is an interesting observation in the context of network theory: if a networked system (here: a university) is characterized by a significantly high number of attractive nodes (here: relatively highly cited publications), then there is ‘less to be preferred’ (because most of the nodes ‘are attractive’) and thus the mechanism of preferential attachment will be less strong.

**Figure 13:**
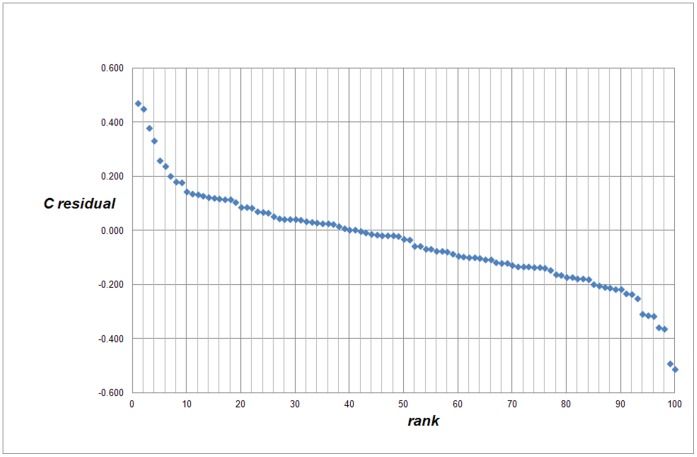
Ranking of the C-residuals of the top-100 European universities.

We calculated the C-residuals (eq.10) and in Fig. 13 we show the ranking of these C-residuals for the top-100 European universities.
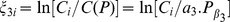
(10)


We notice that the range of the C-residuals in the case of the top-100 European universities (between +0.5 and −0.5) is considerably smaller than in the case of the 500 universities (between +1.0 and −1.5). This difference can be explained by the fact that the top-100 European universities are a quality- (citation impact) based selection within the entire set of 500 universities.

**Figure 14:**
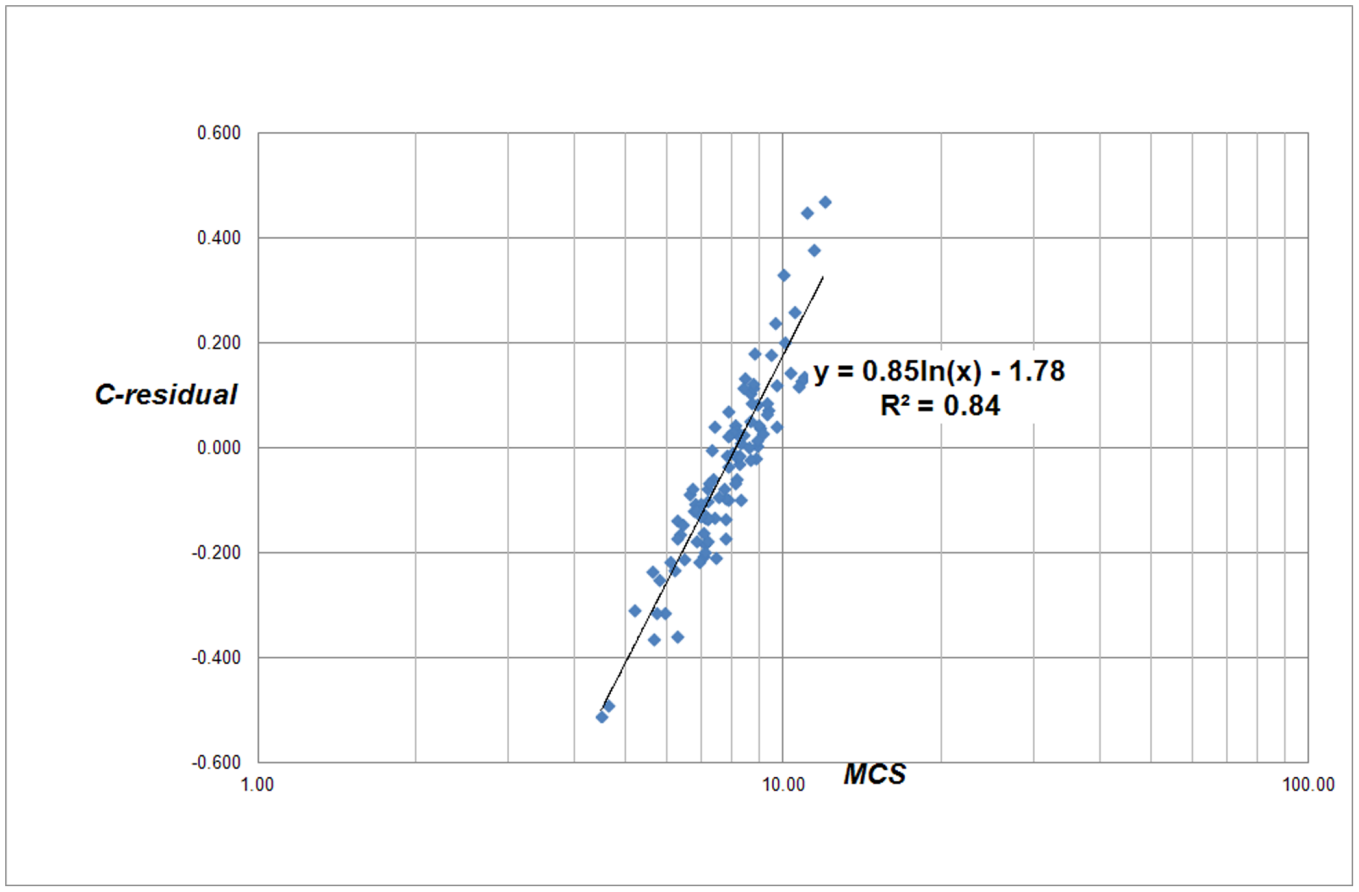
Correlation of the C-residuals with the average number of citations per paper for each university (*MCS*) for the top-100 European universities.

**Figure 15:**
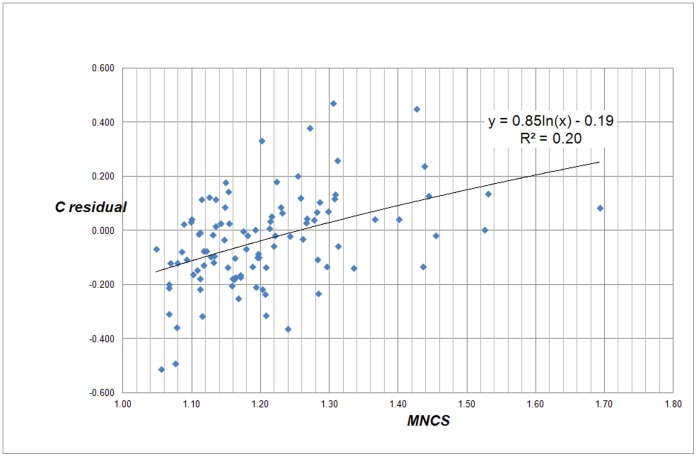
Correlation of the C-residuals with the average number of citations per paper for each university (*MNCS*) for the top-100 European universities.

The correlation of C-residuals with the average number of citations per paper (*MCS*) of a university and the average field-normalized number of citations (*MNCS*) of a university is shown in Figs. 14 and 15.

The correlation of the C-residuals of the top-100 European universities with *MCS* is significantly strong. As discussed earlier, this is to be expected. This correlation however does not significantly follow a logarithmic and can be fit with a linear relation as well. In complete contrast to our findings for the 500 universities we see in Fig. 15 that the correlation of the C-residuals with *MNCS* is hardly significant. This absence of a significant correlation with the *MNCS* indicator is what we can expect: the top-universities are all selected on the basis of high impact research and so this indicator will in this specific case hardly discriminate between these universities.

**Figure 16:**
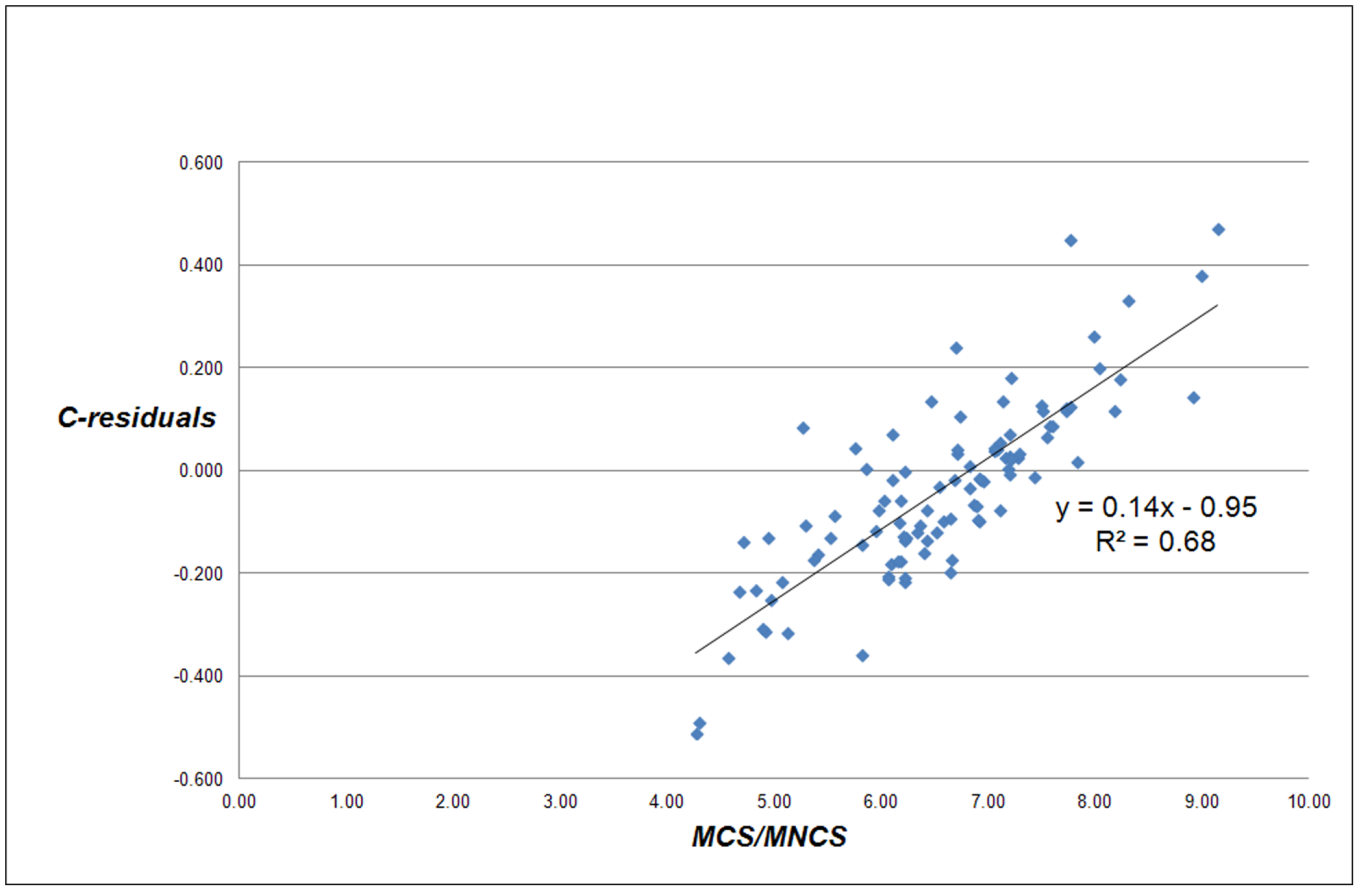
Correlation of the C-residuals with the average field citation-density for each university (*MCS/MNCS*) for the top-100 European universities.

As in the case of the 500 universities, we analyzed the relation between the C-residuals and the average field citation density of a university *MCS*/*MNCS*, see Fig. 16. We see a quite significant relation which again implies that the C-residuals are related with the average field citation-density and thus with the field composition of a university. Indeed, also for the top-100 European universities we find by inspecting the C-residuals of the individual universities, that universities with a large medical school generally have positive C-residuals. Universities with a focus on engineering or social sciences generally have negative C-residuals.

In order to find a relation between the C-residuals and other bibliometric indicators we investigated their relation with (1) the percentage of publications in collaboration, (2) the percentage of publications in international collaboration, and (3) the percentage of publications in long distance collaboration (these indicators are optional parts of the Leiden Ranking [20]). We find that there is no significant relation between the C-residuals and any of these scientific collaboration indicators.

**Figure 17:**
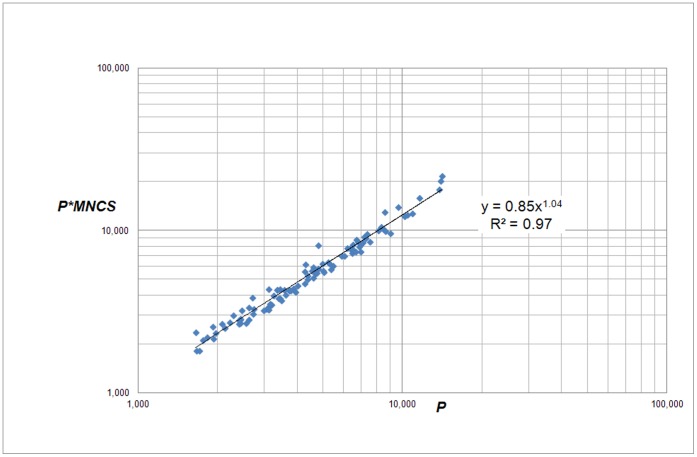
Correlation of *C*_n_ ( =  P*MNCS, the field-normalized number of citations) with *P* for the top-100 European universities.

Like in the case of the 500 universities, we also analyzed the correlation of the field-normalized absolute number of citations (*C*_n_ = P*MNCS) with the absolute number of publications (*P*). The results are shown in Fig. 17. We find a power law relation given by
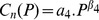
(11)with exponent *β_4_* = 1.04 and coefficient *a* ***_4_*** = 0.85. This exponent is considerably lower than in the case of the scaling of citations with publications and even more, this power law exponent is almost 1 so that in this case we cannot speak of ‘advantage’ of size. This is remarkable contrast with the 500 universities where the exponent of the correlation between P*MNCS with *P* is 1.17 and thus a nonlinear, cumulative advantage by size is present. Evidently, the size-dependent cumulative advantage mechanism is considerably weaker in the case of high impact universities.

In order to avoid the paper becoming lengthy by presenting all further similar details of the B-residuals as in the above analyses, we restrict ourselves to the following observations. The B-residuals of the top-100 European universities do not show a correlation with *MCS* (in the case of the 500 universities there is a reasonable correlation); top-universities generally score high on *MCS* and thus this indicator discriminates only to a small extent between the top-universities. The B-residuals correlate stronger with *MNCS* than in the case of the 500 universities. As discussed earlier, the B-residuals are a function of the real number of field-normalized citations and the real number of publications of a university, and the same is true for *MNCS*. Furthermore, the power exponent of the correlation of P*MNCS with *P* is 1.04, so very close to one, which will make the correlations of the B-residuals and *MNCS* almost perfect. As opposed to the 500 universities, the B-residuals of the top-100 European universities show only a very weak to hardly any correlation with the C-residuals.

### Conclusions

In this paper we demonstrate that universities show a similar behavior as cities in the distribution of the ‘gross university income’ in terms of total number of citations over ‘size’ in terms of total number of publications. Moreover, the power law exponents for university scaling are comparable to those for urban scaling. Also similar to urban scaling, the deviations from the expected behavior can be explained by specific local properties of universities, particularly the field-specific composition of a university, and its quality in terms of field-normalized citation impact.

By studying both the set of the 500 largest universities worldwide and a specific subset of these 500 universities namely the top-100 European universities we are able to distinguish between properties of universities with as well as without selection of one specific local property, the quality of a university in terms of its average field-normalized citation impact. We find a reasonably significant relation of the C-residuals with the average *field-normalized* number of citations of a university (*MNCS*) in the case of the 500 largest universities worldwide, and hardly a significant relation in the case of the top-100 European universities. This implies that the C-residuals are related with the quality (impact) of a university in the case that the set of universities under study is not selected on the basis of quality. If this is the case, such as the top-100 universities, it is obvious that the dependence of the residuals on quality largely disappears.

We also find a reasonably significant relation of the C-residuals with the average field-citation density of a university (*MCS*/*MNCS*), and hence a measure of its field composition, which implies that the C-residuals are related with the average field citation-density and thus with the field composition of a university. We observe this relation in both the set of the 500 largest universities worldwide and in the subset of the top-100 European universities. The analyses of the B-residuals clearly confirm confirms the role of quality as (one of ) the causes for the deviation of universities from the average (expected) scaling behavior.

In summary, our results show that the C-residuals as a measure of deviation from the average scaling behavior relate to two crucial ‘local properties’ of a university.

First, the *citation density of the fields* covered by a university, and thus the field specific composition of a university. For instance, the negative residuals (fewer citations than expected) are characteristic for universities of technology and universities with large engineering departments. And the opposite, the positive residuals are typical for universities with large medical schools. Second, the residuals also relate to the *quality* of a university measured in terms of overall field-normalized citation impact.

Finally, our comparison of sets of universities with and without selection on the basis of citation impact reveals an interesting observation concerning the working of a crucial property in networked systems, preferential attachment. If a networked system (here: a university) is characterized by a significantly high number of attractive nodes (here: relatively highly cited publications), then there is ‘less to be preferred’ (because most of the nodes ‘are attractive’) and thus the mechanism of preferential attachment will be less strong.
